# Combined Impacts of Genetic Variants of Long Non-Coding RNA MALAT1 and the Environmental Carcinogen on the Susceptibility to and Progression of Oral Squamous Cell Carcinoma

**DOI:** 10.3389/fonc.2021.684941

**Published:** 2021-06-29

**Authors:** Yi-Fang Ding, Yu-Ching Wen, Chun-Yi Chuang, Chiao-Wen Lin, Yi-Chieh Yang, Yu-Fan Liu, Wei-Min Chang, Lun-Ching Chang, Shun-Fa Yang, Ming-Hsien Chien

**Affiliations:** ^1^ Graduate Institute of Medical Sciences, College of Medicine, Taipei Medical University, Taipei, Taiwan; ^2^ Department of Otolaryngology, Wan Fang Hospital, Taipei Medical University, Taipei, Taiwan; ^3^ Department of Urology, Wan Fang Hospital, Taipei Medical University, Taipei, Taiwan; ^4^ Department of Urology, School of Medicine, College of Medicine, Taipei Medical University, Taipei, Taiwan; ^5^ School of Medicine, Chung Shan Medical University, Taichung, Taiwan; ^6^ Department of Otolaryngology, Chung Shan Medical University Hospital, Taichung, Taiwan; ^7^ Institute of Oral Sciences, Chung Shan Medical University, Taichung, Taiwan; ^8^ Department of Dentistry, Chung Shan Medical University Hospital, Taichung, Taiwan; ^9^ Graduate Institute of Clinical Medicine, College of Medicine, Taipei Medical University, Taipei, Taiwan; ^10^ Department of Medical Research, Tungs’ Taichung MetroHarbor Hospital, Taichung, Taiwan; ^11^ Department of Biomedical Sciences, College of Medicine Sciences and Technology, Chung Shan Medical University, Taichung, Taiwan; ^12^ School of Oral Hygiene, College of Oral Medicine, Taipei Medical University, Taipei, Taiwan; ^13^ Department of Mathematical Sciences, Florida Atlantic University, Boca Raton, FL, United States; ^14^ Institute of Medicine, Chung Shan Medical University, Taichung, Taiwan; ^15^ Department of Medical Research, Chung Shan Medical University Hospital, Taichung, Taiwan; ^16^ Pulmonary Research Center, Wan Fang Hospital, Taipei Medical University, Taipei, Taiwan; ^17^ Traditional Herbal Medicine Research Center, Taipei Medical University Hospital, Taipei, Taiwan; ^18^ Taipei Medical University (TMU) Research Center of Cancer Translational Medicine, Taipei Medical University, Taipei, Taiwan

**Keywords:** oral squamous cell carcinoma, MALAT1, single-nucleotide polymorphisms, susceptibility, progression

## Abstract

Oral squamous cell carcinoma (OSCC) is the most common malignant tumor of the oral cavity, and long non-coding (lnc)RNA of metastasis-associated lung adenocarcinoma transcript 1 (MALAT1) was recently reported to play a crucial role in OSCC development and progression. However, potential effects of genetic variants of MALAT1 on the development of OSCC are still unclear. Herein, we performed a case-control study in 1350 patients with OSCC and 1199 healthy controls to evaluate the association between functional single-nucleotide polymorphisms (SNPs) of MALAT1 and OSCC susceptibility, as well as its clinicopathologic characteristics. A TaqMan allelic discrimination assay was used to genotype four tagging SNPs, viz., rs3200401 C>T, rs619586 A>G, rs1194338 C>A, and rs7927113 G>A, and results showed that the MALAT1 rs3200401 T allele had a lower risk of OSCC (adjusted odds ratio (AOR): 0.779, 95% confidence interval (CI): 0.632~0.960, *p*=0.019) and a higher risk of developing moderately (grade II)/poorly (grade III) differentiated OSCC (AOR: 1.508-fold, 95% CI: 1.049~2.169, *p*=0.027) under a dominant model. According to environmental carcinogen exposure, patients with a betel quid-chewing habit who carried the T allele of rs3200401 more easily developed high-grade (II/III) OSCC (AOR: 1.588, 95% CI: 1.055~2.390, *p*=0.027), and patients with the same genotype but who did not chew betel quid had a lower risk of developing lymph node metastasis (AOR: 0.437, 95% CI: 0.255~0.749, *p*=0.003). In addition to rs3200401, the rs619586 AG/GG genotype was associated with increased risks of developing advanced stages (III+IV) and larger tumor sizes (>T2) compared to the AA genotype, especially in the subgroup of betel quid chewers. Furthermore, analyses of clinical datasets revealed that the MALAT1 expression level was upregulated in OSCC compared to normal tissues, especially in the betel quid-chewing population. These results indicated involvement of MALAT1 SNPs rs3200401 and rs619586 in the development of OSCC and support the interaction between MALAT1 gene polymorphisms and the environmental carcinogen as a predisposing factor for OSCC progression.

## Introduction

Oral squamous cell carcinoma (OSCC) is one of the six most frequent cancers in the world, the causes of OSCC are complex, and a lot of factors contribute to its development and progression. For example, continuous exposure to tobacco, alcohol use, and human papillomavirus infection are common, major risk factors for OSCC worldwide (1, 2), and betel nut chewing is another predominant risk factor causing OSCC in Taiwan (3). Until now, the pathogenesis of OSCC has remained unclear, but it was generally reported that OSCC is attributed to combined effects of various risk factors and genetic and epigenetic changes. Although great advances have been made in treating OSCC, including surgery, chemotherapy, and radiotherapy, the 5-year survival rate is only 50% (4), mainly due to delays in diagnoses which allows the cancer to metastasize. Thus, finding notable prognostic factors, metastatic predictors, and therapeutic targets in OSCC is urgently needed.

Long non-coding (lnc)RNAs are a type of RNA molecule with a length of >200 nucleotides (nt), which are unable to encode proteins. Expanding evidence indicates that lncRNAs are notable molecular markers involved in modulating gene expressions and cancer progression, such as tumor cell proliferation, invasion, metastasis, and angiogenesis (5–7). Metastasis-associated lung adenocarcinoma transcript 1 (MALAT1) is one of the most widely studied nuclear-retained lncRNAs that has garnered much attention in recent years due to its abundance and apparent role in various diseases. MALAT1 was shown to act as a competing endogenous (ce)RNA or micro (mi)RNA sponge which sequesters miRNAs under various conditions (8). In terms of cancer, MALAT1 was initially identified as an RNA whose expression is upregulated in primary lung tumors that had higher metastatic abilities (9). In addition to lung cancer, pro-oncogenic and prometastatic roles of MALAT1 were reported in a wide range of solid and non-solid tumors including OSCC. For instance, MALAT1 can function as a ceRNA to modulate signal transduction and activator of transcription 3 (STAT3) expression by absorbing miR-125b and further promote the growth of OSCC (10). The miR-101/enhancer of zeste homolog 2 (EZH2) axis is another pathway regulated by MALAT1 to facilitate proliferation and invasion of OSCC cells (11).

Recently, increasing evidence has indicated that single-nucleotide polymorphisms (SNPs) are universally present in lncRNA genes, and they may directly or indirectly influence lncRNA expression levels through various means and thus are likely to regulate the development and progression of cancer (12). To date, a number of SNPs in lncRNAs were found to be related to the development, progression, and prognosis of OSCC such as *maternally expressed 3* (*MEG3*) (13), *phosphatase and tensin homolog pseudogene 1* (*PTENP1*) (14), *H19* (15), *HOX transcript antisense RNA* (*HOTAIR*) (16), and so on. Although MALAT1 SNPs such as rs619586 were reported to be associated with the risk or progression of several cancer types and expression of MALAT1 (17), little is known about the effects of polymorphisms of MALAT1 on the development and progression of OSCC, especially in Asian populations. In the present study, a case-control study in a Taiwanese population was performed to identify roles of MALAT1 SNPs in the risk and clinical characteristics of OSCC.

## Materials and Methods

### Selection of Study Subjects

In total, 1350 male patients with OSCC treated at Chung Shan Medical University Hospital (Taichung, Taiwan) between 2007 and 2019 were recruited for this study. All participants provided informed consent. In total, 1199 anonymized healthy controls were randomly selected from the Taiwan Biobank Project; none had a previous history of cancer at any site. Moreover, subjects with oral precancerous disease, including oral submucosal fibrosis, leukoplakia, erythroplakia, verrucous hyperplasia, etc., were excluded from the control group. A questionnaire was completed by all participants *via* face-to-face interviews to obtain information about the patient’s exposure to betel quid chewing, tobacco use, and alcohol consumption. Medical information of OSCC patients was obtained from their medical records including tumor, node, metastasis (TNM) clinical staging, the primary tumor size, lymph node involvement, distal metastasis, and histologic grade. This study was approved by the Ethics Committee of Chung Shan Medical University Hospital (no. CS15125).

### Cell Lines and Culture

HSC3 and SAS cell lines were obtained from Japanese Collection of Research Bioresources (JCRB) Cell Bank (Osaka, Japan) and cultured in Dulbecco’s Modified Eagle Medium/Nutrient Mixture F-12 (DMEM/F12; Life Technologies, Grand Island, NY). CAL27 cell line was obtained from American Type Culture Collection (ATCC) (Manassas, VA, USA) and cultured in DMEM medium (Life Technologies). In addition, OECM1 cell line was cultured in RPMI-1640 medium (Life Technologies). All cell culture media were all supplemented with 10% fetal bovine serum (FBS) (Gibco, Grand Island, NY) and all the cells were maintained at 37°C in a humidified atmosphere of 5% CO_2_.

### Genomic DNA Extraction and MALAT1 SNP Selection

**Figure 1:**
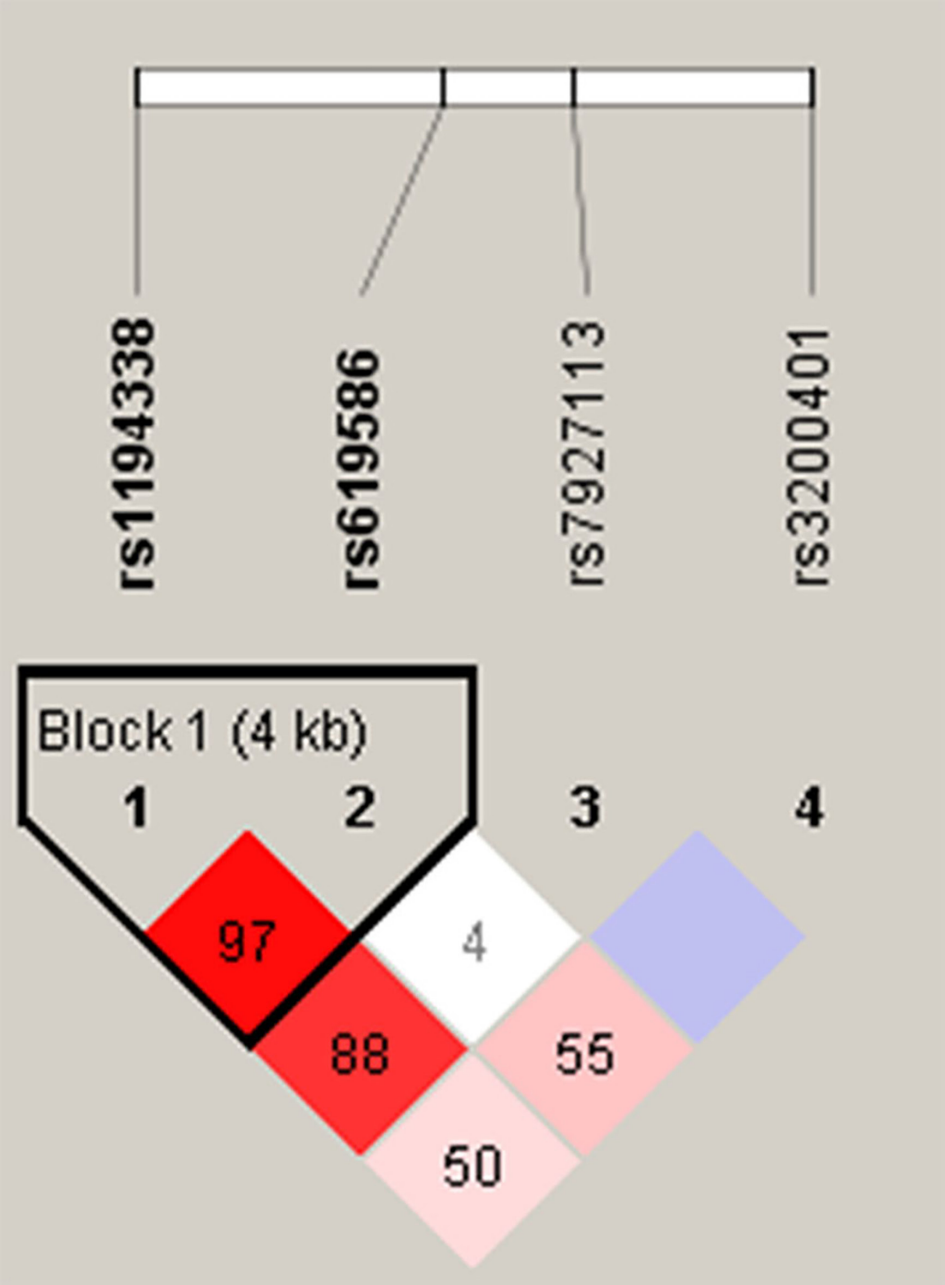
Linkage disequilibrium (LD) map for single nucleotide polymorphisms in the *MALAT1* gene. Block is pairwise D’ plots and haplotype blocks obtained from HAPLOVIEW.

Venous blood of all participants was placed in tubes containing ethylenediaminetetraacetic acid (EDTA) and further centrifuged at 3000 rpm for buffy coat isolation. DNA from blood leukocytes or OSCC cells was further extracted using a QIAamp DNA Mini Kit (Qiagen, Valencia, CA, USA) according to the manufacturer’s instructions. The purity and concentration of DNA were determined with a Nanodrop-2000 spectrophotometer (Thermo Fisher Scientific, Waltham, MA, USA), with DNA stored at -20°C before genotyping. TagSNPs of lncRNA MALAT1 were selected based on data from the National Center for Biotechnology Information (NCBI) dbSNP database (http://www.ncbi.nlm.nih.gov/) (18) and International HapMap Project (http://hapmap.org). As a result three tagging SNPs (rs3200401, rs7927113, and rs619586) in MALAT1 were selected, which were representative and could capture all other common SNPs. We also included another novel MALAT1 promoter SNP, rs1194338, which was recently reported to be associated with a risk of colorectal carcinoma in a Han Chinese population (19). The reconstructed linkage disequilibrium (LD) plot of these four SNPs is shown in **Figure 1**. We determined one observed haploblock in which rs1194338 was in strong LD with rs619586 in our study. Most importantly, these four SNPs were selected for this study since they were reported to affect risk or progression of various cancer types in the Han Chinese population (20, 21).

### Genotyping of MALAT1 SNPs

Candidate SNPs including rs3200401 (assay ID: C_3246069_10), rs619586 (assay ID: C_1060479_10), rs1194338 (assay ID: C_11661801_10), and rs7927113 (assay ID: C_ 29370554_10) were genotyped with a TaqMan SNP Genotyping Assay on an ABI StepOnePlus™ Real-Time PCR platform (Thermo Fisher Scientific). The final collected data were further analyzed with ABI StepOnePlus™ Software v2.3 (Applied Biosystems, Foster City, CA).

### Bioinformatics Analysis

Transcriptomic data from Taiwanese samples of adjacent non-tumor tissues and OSCC tissues (*n*=40) were analyzed using microarray datasets with accession number GSE37991 obtained from the Gene Expression Omnibus (GEO) database (https://www.ncbi.nlm.nih.gov/geo/) to investigate whether MALAT1 expression (probe ID: ILMN_2141650) was associated with OSCC tumor formation. Gene expression levels of MALAT1 in normal tissues and head and neck cancerous tissues from Americans were obtained and analyzed from The Cancer Genome Atlas (TCGA)-HNSC database, which was downloaded from the UCSC Xena browser (https://xenabrowser.net/).

### Predicting the Structure of the MALAT1/miRNA Duplex

Based on the lncRNASNP2 database (http://bioinfo.life.hust.edu.cn/lncRNASNP2) and the criterion of a minor allele frequency (MAP) of >5% in Asian populations, the targeted rs619586 SNP, NC_000011.10:g.65498698 A>G, was searched in MALAT1 lncRNA. Search results in the section of SNP caused the miRNA target gain represented three miRNAs candidates including hsa-miR-3619-5p, hsa-miR-761 and hsa-miR-214-3p. We focused on the short transcript form (NR_002019.4, NONHSAT022125.2) of MALAT1 lncRNA for predicting potential lncRNA-miRNA interactions. The minimum free energy (MFE) are -19.5, -22.6 and -18.8 kcal/mol calculated by BiBiserv2 RNAhybrid that G variant on MALAT1 lncRNA could induce target interaction sites gain from miRNAs of hsa-miR-3619-5p, hsa-miR-761 and hsa-miR-214-3p, respectively.

#### RNA Preparation and SYBR Green Quantitative Real-Time Polymerase Chain Reaction (qRT-PCR)

Total RNA was isolated from OSCC cells using Total RNA mini kit (Geneaid Biotech Ltd, New Taipei City, Taiwan). Reverse transcription was provided in the High-Capacity cDNA Reverse Transcription Kit (Termo Fisher Scientifc, Waltham, MA, USA), and mRNA expression was detected by qRT-PCR analysis using SYBR Green Fast qPCR System (Termo Fisher Scientifc). GAPDH was selected as the internal reference. The primer sequences were designed as follows: GAPDH-forward (5’-GGAGCGAGATCCCTCCAAAAT-3’), GAPDH-reverse (5’-GGCTGTTGTCATACTTCTCATGG-3’), MALAT1-forward (5’-GATTGAGGAGGCTGTGCTGT-3’), MALAT1-reverse (5’- CAGCTGCCTGCTGTTTTCTG-3’), CTNNB1-forward (5’-GTGCTATCTGTCTGCTCTAGTA-3’), CTNNB1-reverse (5’-CTTCCTGTTTAGTTGCAGCATC-3’).

### Statistical Analysis

Differences in demographic variables between the cancer-free (control) and OSCC groups were analyzed by the Mann-Whitney *U*-test and Fisher’s exact test. The adjusted odds ratios (AORs) and 95% confidence intervals (CIs) of associations of different MALAT1 SNP distributions with OSCC risk or clinical pathological characteristics of OSCC were estimated by multiple logistic regression models after controlling for other covariates, including age, betel quid chewing, cigarette smoking, and alcohol consumption. All statistics were performed using SAS statistical software (vers. 9.1, 2005; SAS Institute, Cary, NC, USA), and *p*<0.05 was considered statistically significant.

## Results

### Characteristics of Study Participants

Demographic and lifestyle characteristics of the 1350 OSCC cases and 1199 cancer-free controls enrolled in our study were first compared, and results are shown in **Table 1**. The age distributions between the control group and case group were similar. Consistent with previous studies of OSCC patients in Asia (22, 23), higher frequencies of betel nut chewing (*p*<0.001), alcohol consumption (*p*<0.001), and tobacco use (*p*<0.001) were observed in OSCC patients compared to the control group, indicating that these lifestyle characteristics might be critical in the pathogenesis of oral carcinogenesis. The majority of OSCC cases exhibited no lymph node invasion (65.9%) or distal metastasis (99.3%), and their tumors were graded as moderately/poorly differentiated (86.1%).

**Table 1:** Distributions of demographic characteristics in 1199 healthy controls and 1350 male patients with oral cancer.

Variable	Controls (*N*=1199)	Patients (*N*=1350)	*p* value
Age (years)			
≤55	610 (50.9%)	681 (50.4%)	*p*=0.828
>55	589 (49.1%)	669 (49.6%)	
Betel quid chewing			
No	1000 (83.4%)	343 (25.4%)	
Yes	199 (16.6%)	1007 (74.6%)	*p*<0.001*
Cigarette smoking			
No	564 (47.0%)	210 (15.6%)	
Yes	635 (53.0%)	1140 (84.4%)	*p*<0.001*
Alcohol consumption			
No	962 (80.2%)	712 (52.7%)	
Yes	237 (19.8%)	638 (47.3%)	*p*<0.001*
Stage			
I+II		634 (47.0%)	
III+IV		716 (53.0%)	
Tumor T status			
T1+T2		686 (50.8%)	
T3+T4		664 (49.2%)	
Lymph node status			
N0		890 (65.9%)	
N1+N2+N3		460 (34.1%)	
Metastasis			
M0		1340 (99.3%)	
M1		10 (0.7%)	
Cell differentiation			
Well differentiated		188 (13.9%)	
Moderately or poorly differentiated		1162 (86.1%)	

The Mann-Whitney U-test or Fisher’s exact test was used for comparisons between healthy controls and patients with oral cancer. *Statistically significant at p < 0.05.

### Associations Between MALAT1 Genetic Polymorphisms and OSCC Risks

To determine associations of selected MALAT1 SNPs (rs3200401, rs619586, rs1194338, and rs7927113) with OSCC in this Taiwanese population, we utilized AORs (with 95% CIs) which were estimated by multiple logistic regression models after adjusting for other variables (age, betel nut chewing, cigarette smoking, and alcohol consumption), together with the OR (with 95% CI) of each comparison. As shown in **Table 2**, distributions of MALAT1 genotypes revealed that the most frequent alleles were homozygous C/C, A/A, and G/G for the rs3200401, rs619586, and rs7927113 loci, respectively, and heterozygous C/A for the rs1194338 locus. We observed that subjects with MALAT1 polymorphic rs3200401 T/T and a combination of the C/T and T/T genotypes respectively exhibited significantly lower risks of 0.548- (95% CI: 0.319~0.940) and 0.779-fold (95% CI: 0.632~0.960) of having OSCC compared to those with the C/C wild-type (WT) homozygotes.

**Table 2:** Odds ratio (OR) and 95% confidence interval (CI) of oral cancer associated with *MALAT1* genotypic frequencies.

Variable	Controls (N = 1199) (%)	Patients (N = 1350) (%)	OR (95% CI)	AOR (95% CI)^a^
**rs3200401**				
CC	807 (67.3%)	948 (70.2%)	1.000 (reference)	1.000 (reference)
CT	347 (28.9%)	363 (26.9%)	0.890 (0.748-1.060)	0.872 (0.654-1.010) *p* = 0.061
TT	45 (3.8%)	39 (2.9%)	0.737 (0.475-1.144)	**0.548 (0.319-0.940)** ***p* = 0.029**
CT+TT	392 (32.7%)	402 (29.8%)	0.873 (0.738-1.032)	**0.779 (0.632-0.960)** ***p* = 0.019**
**rs619586**				
AA	1015 (84.7%)	1135 (84.1%)	1.000 (reference)	1.000 (reference)
AG	177 (14.8%)	202 (15.0%)	1.021 (0.820-1.270)	0.995 (0.760-1.304) *p* = 0.973
GG	7 (0.5%)	13 (0.9%)	1.661 (0.660-4.179)	1.113 (0.346-3.578) *p* = 0.858
AG+GG	184 (15.3%)	215 (15.9%)	1.045 (0.843-1.295)	1.000 (0.767-1.304) *p* = 0.998
**rs1194338**				
CC	505 (42.1%)	588 (43.6%)	1.000 (reference)	1.000 (reference)
CA	544 (45.4%)	625 (46.3%)	0.987 (0.836-1.164)	1.009 (0.822-1.238) *p* = 0.934
AA	150 (12.5%)	137 (10.1%)	0.784 (0.605-1.018)	0.729 (0.527-1.008) *p* = 0.056
CA+AA	694 (57.9%)	762 (56.4%)	0.943 (0.806-1.104)	0.946 (0.779-1.150) *p* = 0.579
**rs7927113**				
GG	1191 (99.3%)	1338 (99.1%)	1.000 (reference)	1.000 (reference)
GA	8 (0.7%)	12 (0.9%)	1.335 (0.544-3.277)	0.932 (0.297-2.926) *p* = 0.904
AA	-	-	-	-
GA+AA	8 (0.7%)	12 (0.9%)	1.335 (0.544-3.277)	0.932 (0.297-2.926) *p* = 0.904

The odds ratio (OR) with their 95% confidence intervals were estimated by logistic regression models.

a The adjusted odds ratio (AOR) with their 95% confidence intervals were estimated by multiple logistic regression models after controlling for age, betel quid chewing, cigarette smoking, and alcohol drinking. Bold values mean the p value is significant (p < 0.05).

### Relationships of Clinicopathological Characteristics With MALAT1 Genetic Polymorphisms in OSCC Patients

Since MALAT1 genetic polymorphisms were found to be correlated with susceptibility to OSCC, we further explored the effects of MALAT1 SNPs on the clinical status of OSCC patients, such as the clinical stage, primary tumor size, lymph node involvement, metastatic status, and histopathologic grading. We found that patients with at least one minor allele (CT or TT) of rs3200401 exhibited a significantly higher risk of developing moderately (grade II)/poorly (grade III) differentiated OSCC (AOR: 1.508-fold; 95% CI: 1.049~2.169; *p*=0.027) compared to their counterparts with the corresponding WT homozygotes (**Table 3**). In addition to rs3200401, MALAT1 rs619586 polymorphisms presented significant differences in terms of clinical stage (AOR: 1.358-fold; 95% CI: 1.009~1.827; *p*=0.044) and tumor size (AOR: 1.429-fold; 95% CI: 1.064~1.919; *p*=0.018) in OSCC patients with at least one minor allele (AG or GG) (**Table 4**).

**Table 3:** Adjusted odds ratios (AORs) and 95% confidence intervals (CIs) of clinical statuses associated with genotypic frequencies of *MALAT1* rs3200401 in male oral cancer patients (*N*=1350).

Variable			AOR (95% CI)	*p* value
	**Clinical stage**			
**rs3200401**	Stage I+II (*n*=634) (%)	Stage III+IV (*n*=716) (%)		
CC	429 (67.7%)	519 (72.5%)	1.00	
CT+TT	205 (32.3%)	197 (27.5%)	0.796 (0.630~1.007)	*p*=0.057
	**Tumor size**			
**rs3200401**	≤T2 (*n*=686) (%)	>T2 (*n*=664) (%)		
CC	484 (70.5%)	464 (69.9%)	1.00	
CT+TT	202 (29.5%)	200 (30.1%)	1.023 (0.809~1.293)	*p*=0.849
	**Lymph node metastasis**			
**rs3200401**	No (*n*=890) (%)	Yes (*n*=460) (%)		
CC	613 (68.9%)	335 (72.8%)	1.00	
CT+TT	277 (31.1%)	125 (27.2%)	0.829 (0.645~1.066)	*p*=0.143
	**Metastasis**			
**rs3200401**	M0 (*n*=1340) (%)	M1 (*n*=10) (%)		
CC	942 (70.3%)	6 (60.0%)	1.00	
CT+TT	398 (29.7%)	4 (40.0%)	1.619 (0.452~5.795)	*p*=0.459
	**Cell differentiation grade**			
**rs3200401**	≤Grade I (*n*=188) (%)	>Grade I (*n*=1162) (%)		
CC	145 (77.1%)	803 (69.1%)	1.00	
CT+TT	43 (22.9%)	359 (30.9%)	**1.508 (1.049~2.169)**	*p*=0.027^*^

Cell differentiation grade: grade I, well differentiated; grade II, moderately differentiated; grade III, poorly differentiated.

The AORs with their 95% CIs were estimated by multiple logistic regression models after controlling for age, betel quid chewing, cigarette smoking, and alcohol consumption. *Statistically significant at p < 0.05. Bold values mean the p value is significant (p < 0.05).

**Table 4:** Adjusted odds ratios (AORs) and 95% confidence intervals (CIs) of clinical statuses associated with genotypic frequencies of *MALAT1* rs619586 in male oral cancer patients (*N*=1350).

Variable			AOR (95% CI)	*p* value
	**Clinical stage**			
**rs619586**	Stage I+II (*n*=634) (%)	Stage III+IV (*n*=716) (%)		
AA	546 (86.1%)	589 (82.3%)	1.00	
AG+GG	88 (13.9%)	127 (17.7%)	**1.358 (1.009~1.827)**	*p*=0.044^*^
	**Tumor size**			
**rs619586**	≤T2 (*n*=686) (%)	>T2 (*n*=664) (%)		
AA	593 (86.4%)	542 (81.6%)	1.00	
AG+GG	93 (13.6%)	122 (18.4%)	**1.429 (1.064~1.919)**	*p*=0.018^*^
	**Lymph node metastasis**			
**rs619586**	No (*n*=890) (%)	Yes (*n*=460) (%)		
AA	748 (84.0%)	387 (84.1%)	1.00	
AG+GG	142 (16.0%)	73 (15.9%)	1.005 (0.738~1.370)	*p*=0.973
	**Metastasis**			
**rs619586**	M0 (*n*=1340) (%)	M1 (*n*=10) (%)		
AA	1125 (84.0%)	10 (100.0%)	1.00	
AG+GG	215 (16.0%)	0 (40.0%)	-	-
	**Cell differentiation grade**			
**rs619586**	≤Grade I (*n*=188) (%)	>Grade I (*n*=1162) (%)		
AA	159 (84.6%)	976 (84.0%)	1.00	
AG+GG	29 (15.4%)	186 (16.0%)	1.043 (0.681~1.599)	*p*=0.846

Cell differentiation grade: grade I, well differentiated; grade II, moderately differentiated; grade III, poorly differentiated.

The AORs with their 95% CIs were estimated by multiple logistic regression models after controlling for age, betel quid chewing, cigarette smoking, and alcohol consumption. *Statistically significant at p < 0.05. Bold values mean the p value is significant (p < 0.05).

### Stratified Analysis of MALAT1 Genetic Associations With Clinicopathological Characteristics of OSCC Patients

Actually, chewing betel quid was reported to be the strongest risk factors for causing OSCC in East Asian population (24). Taiwan is also an endemic betel quid-chewing area, and betel quid chewing was reported to be correlated with a poor prognosis in OSCC (25). In this study, we further divided our recruited OSCC patients into betel quid-chewing and non-betel quid-chewing groups, and further investigated the difference between MALAT1 SNPs and the OSCC clinicopathological status in these two subgroups. Compared to the overall OSCC group, development of advance clinical (III+IV) and tumor T stage (>T2) was further strengthened in the betel quid-chewing subgroup who harbored at least one polymorphic G allele of MALAT1 rs619586 (**Table 5**). The risk of developing high-grade OSCC was further heightened in individuals who chewed betel nut and also carried at least one mutant allele of rs3200401 (**Table 6**). In contrast to the betel quid-chewing subgroup, OSCC patients with at least one T allele of MALAT1 rs3200401 were at a lower risk for developing lymph node metastasis if they did not chew betel nuts (**Table 7**). These results suggested that a potential interaction between betel nut chewing and the existence of at least one polymorphic allele of these two MALAT1 SNPs was shown to be correlated with OSCC progression. In addition to betel quid-chewing, two other common environmental carcinogens-alcohol consumption and tobacco use-were also selected to investigate their impacts with MALAT1 SNPs on the clinicopathological status of OSCC. We observed that OSCC patients with the smoking or drinking habit who had at least one G allele of MALAT1 rs619586 were at higher risk of developing advance clinical stage and larger tumor sizes compared to those patients with AA homozygotes (**Supplementary Tables 1**, **2**). Moreover, OSCC patients with the smoking or drinking habit who had at least one T allele of MALAT1 rs3200401 were at higher risk of developing high-grade OSCC compared to those patients with CC homozygotes (**Supplementary Tables 3**, **4**). Compared to the overall OSCC group, the risk of OSCC within advanced-stage development were strengthened in the alcohol consumption or tobacco use subgroups who harbored at least one polymorphic G allele of MALAT1 rs619586 or T allele of MALAT1 rs3200401 (**Supplementary Tables 1**–**4**).

**Table 5:** Adjusted odds ratios (AORs) and 95% confidence intervals (CIs) of clinical statuses associated with genotypic frequencies of *MALAT1* rs619586 in male oral cancer patients who chewed betel nuts (*N*=1007).

Variable			AOR (95% CI)	*p* value
	**Clinical stage**			
**rs619586**	Stage I+II (*n*=480) (%)	Stage III+IV (*n*=527) (%)		
AA	414 (86.3%)	430 (81.6%)	1.00	
AG+GG	66 (13.7%)	97 (18.4%)	**1.419 (1.009~1.997)**	*p*=0.045^*^
	**Tumor size**			
**rs619586**	≤T2 (*n*=519) (%)	>T2 (*n*=488) (%)		
AA	448 (86.3%)	396 (81.2%)	1.00	
AG+GG	71 (13.7%)	92 (18.8%)	**1.452 (1.035~2.038)**	*p*=0.031^*^
	**Lymph node metastasis**			
**rs619586**	No (*n*=675) (%)	Yes (*n*=332) (%)		
AA	565 (83.7%)	279 (84.0%)	1.00	
AG+GG	110 (16.3%)	53 (16.0%)	0.972 (0.678~1.391)	*p*=0.875
	**Metastasis**			
**rs619586**	M0 (*n*=1000) (%)	M1 (*n*=7) (%)		
AA	837 (83.7%)	7 (100.0%)	1.00	
AG+GG	163 (16.3%)	0 (0.0%)	-	-
	**Cell differentiation grade**			
**rs619586**	≤Grade I (*n*=151) (%)	>Grade I (*n*=856) (%)		
AA	125 (82.8%)	719 (84.0%)	1.00	
AG+GG	26 (17.2%)	137 (16.0%)	0.901 (0.568~1.429)	*p*=0.657

Cell differentiation grade: grade I, well differentiated; grade II, moderately differentiated; grade III, poorly differentiated.

The AORs with their 95% CIs were estimated by multiple logistic regression models after controlling for age, cigarette smoking, and alcohol consumption. *Statistically significant at p < 0.05. Bold values mean the p value is significant (p < 0.05).

**Table 6:** Adjusted odds ratios (AORs) and 95% confidence intervals (CIs) of clinical statuses associated with genotypic frequencies of *MALAT1* rs3200401 in male oral cancer patients who chewed betel nuts (*N*=1007).

Variable			AOR (95% CI)	*p* value
	**Clinical stage**			
**rs3200401**	Stage I+II (*n*=480) (%)	Stage III+IV (*n*=527) (%)		
CC	326 (67.9%)	374 (71.0%)	1.00	
CT+TT	154 (32.1%)	153 (29.0%)	0.861 (0.658~1.127)	*p*=0.277
	**Tumor size**			
**rs3200401**	≤T2 (*n*=519) (%)	>T2 (*n*=448) (%)		
CC	359 (69.2%)	341 (69.9%)	1.00	
CT+TT	160 (30.8%)	147 (30.1%)	0.960 (0.733~1.256)	*p*=0.764
	**Lymph node metastasis**			
**rs3200401**	No (*n*=675) (%)	Yes (*n*=332) (%)		
CC	470 (69.6%)	230 (69.3%)	1.00	
CT+TT	205 (30.4%)	102 (30.7%)	1.006 (0.756~1.340)	*p*=0.966
	**Metastasis**			
**rs3200401**	M0 (*n*=1000) (%)	M1 (*n*=7) (%)		
CC	696 (69.6%)	4 (57.1%)	1.00	
CT+TT	304 (30.4%)	3 (42.9%)	1.774 (0.393~7.997)	*p*=0.456
	**Cell differentiation grade**			
**rs3200401**	≤Grade I (*n*=151) (%)	>Grade I (*n*=856) (%)		
CC	117 (77.5%)	583 (68.1%)	1.00	
CT+TT	34 (22.5%)	273 (31.9%)	**1.588 (1.055~2.390)**	*p*=0.027^*^

Cell differentiation grade: grade I, well differentiated; grade II, moderately differentiated; grade III, poorly differentiated.

The AORs with their 95% CIs were estimated by multiple logistic regression models after controlling for age, cigarette smoking, and alcohol consumption. *Statistically significant at p < 0.05. Bold values mean the p value is significant (p < 0.05).

**Table 7:** Adjusted odds ratios (AORs) and 95% confidence intervals (CIs) of clinical statuses associated with genotypic frequencies of *MALAT1* rs3200401 in male oral cancer patients who did not chew betel nuts (*N*=343).

Variable			AOR (95% CI)	*p* value
	**Clinical stage**			
**rs3200401**	Stage I+II (*n*=154) (%)	Stage III+IV (*n*=189) (%)		
CC	103 (66.9%)	145 (76.7%)	1.00	
CT+TT	51 (33.1%)	44 (23.3%)	0.618 (0.382~1.001)	*p*=0.051
	**Tumor size**			
**rs3200401**	≤T2 (*n*=167) (%)	>T2 (*n*=176) (%)		
CC	125 (74.9%)	123 (69.9%)	1.00	
CT+TT	42 (25.1%)	53 (30.1%)	1.254 (0.777~2.024)	*p*=0.355
	**Lymph node metastasis**			
**rs3200401**	No (*n*=215) (%)	Yes (*n*=128) (%)		
CC	143 (66.5%)	105 (82.0%)	1.00	
CT+TT	72 (33.5%)	23 (18.0%)	**0.437 (0.255*-*0.749)**	*p*=0.003^*^
	**Metastasis**			
**rs3200401**	M0 (*n*=340) (%)	M1 (*n*=3) (%)		
CC	246 (72.4%)	2 (66.7%)	1.00	
CT+TT	94 (27.6%)	1 (33.3%)	1.324 (0.117~14.989)	*p*=0.821
	**Cell differentiation grade**			
**rs3200401**	≤Grade I (*n*=37) (%)	>Grade I (*n*=306) (%)		
CC	28 (75.7%)	220 (71.9%)	1.00	
CT+TT	9 (24.3%)	86 (28.1%)	1.198 (0.540~2.658)	*p*=0.656

Cell differentiation grade: grade I, well differentiated; grade II, moderately differentiated; grade III, poorly differentiated.

The AORs with their 95% CIs were estimated by multiple logistic regression models after controlling for age, cigarette smoking, and alcohol consumption. *Statistically significant at p < 0.05. Bold values mean the p value is significant (p < 0.05).

### MALAT1 Expression Is Upregulated in OSCC, Especially in Patients With the Habit of Chewing Betel Quid

**Figure 2:**
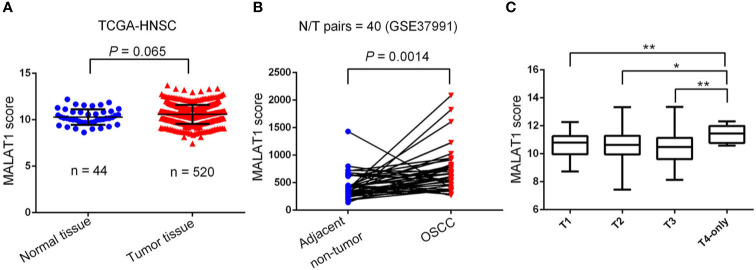
Clinical relevance of metastasis-associated lung adenocarcinoma transcript 1 (MALAT1) levels in head and neck squamous cell carcinomas (HNSCC) patients obtained from TCGA and GEO databases. **(A)** *MALAT1* gene expression levels in normal and HNSCC tissues were compared according to data from TCGA datasets. Statistical significance was analyzed by a *t*-test. **(B)** *MALAT1* gene expression levels in an oral squamous cell carcinoma normal/tumorous (N/T) paired cohort with the habit of betel quid chewing (GSE37991). Statistical significance was analyzed by a paired *t*-test. **(C)** *MALAT1* gene expression levels in HNSCC from TCGA were compared according to the tumor size (T stages). Statistical significance was analyzed by a *t*-test. *p < 0.05, **p < 0.01.

To further dissect expression levels of MALAT1 and their clinical significance in oral cancer, cases of head and neck squamous cell carcinomas (HNSCC) were analyzed from TCGA dataset. Compared to noncancerous tissues, MALAT1 expression was prone to be upregulated in HNSCC (*p*=0.065) (**Figure 2A**). Interestingly, from the GSE37991 dataset of the GEO database, we further observed that MALAT1 expression levels were significantly higher in OSCC specimens than their corresponding matched normal tissues from an OSCC N/T paired Taiwanese cohort with the habit of betel quid chewing (*p*=0.0014) (**Figure 2B**). Moreover, relative levels of MALAT1 transcripts were higher in HNSCC patients with larger tumors (T4 status) than in patients with smaller tumors (T1, T2, or T3 status) (**Figure 2C**).

## Discussion

Recently, lncRNAs were shown to play important roles in cancer development, and a large number of lncRNAs associated with multiple cancers were identified, including OSCC (26, 27). Oncogenic roles of lncRNA MALAT1 in OSCC were previously reported, including promotion of growth, metastasis, and chemoresistance of OSCC through inducing the epithelial-to-mesenchymal transition (EMT) and sponging tumor-suppressive miRNAs such as miR-143, miR-125b, and miR-101 (10, 11, 28–30). However, OSCC risks and clinicopathologic characteristics conferred by genetic variants on loci of MALAT1 have rarely been the focus of epidemiological investigations.

In the present molecular epidemiology study conducted in 1350 OSCC patients and 1199 healthy controls, we found that mutant base T of rs3200401 was significantly associated with a lower risk of OSCC, regardless of the codominant model (TT) or dominant model (CT+TT). Similar to our results, previous studies showed that the risk of prostate cancer (PCa) development in a Ukrainian population was significantly lower in those with the rs3200401 TT genotype compared to CC genotype (31). In breast cancer, the CT genotype of rs3200401 imparted a lower risk of breast cancer compared to CC in a Han Chinese population (20). In addition, Wang et al. indicated that advanced lung cancer patients with the rs3200401 CT and TT genotypes had significantly longer overall survival times than did patients with the CC genotype (32). Volkogon et al. found that bladder cancer patients with the rs3200401 TT genotype had significantly longer disease-free survival times than did patients with the C allele (33). Taken together, results of our study were accordant with previous studies mentioned above and revealed that the T allele of rs3200401 may have a protective role against the development of cancer. Wang et al. showed that the rs3200401 C>T nucleotide replacement led to 1.62 kcal/mol MFE change, which alters MALAT1’s spatial structure and impairs its interaction with serine/arginine-rich splicing factor 2 (SRSF2) (32). Violation of interactions between MALAT1 and SRSF2 ultimately lead to inhibition of pre-mRNA alternative splicing and expressions of genes involved in cancer development, and such an effect might explain both the decreased tumor aggression activity and better survival rates in patients with various cancer types who are minor T-allele carriers (32).

In contrast to the protective effect against malignant tumor development, the rs3200401 TT genotype was reported to increase the risk of esophageal squamous cell carcinoma (ESCC) and tumor width of bladder cancer compared to the rs3200401 CC genotype (34). Our present results showed that OSCC patients with at least one minor allele (CT or TT) of rs3200401 exhibited a significantly higher risk of developing high-grade tumors, especially in the subgroup of betel quid chewers. Detailed mechanisms of the opposite role of rs32000401 polymorphisms in different cancers remain to be identified. We hypothesized that environmental carcinogens might be the potential effector driving the protective or oncogenic role of rs32000401 polymorphisms in cancers, because our study showed that OSCC patients harboring at least one T allele of MALAT1 rs3200401 were at a lower risk of developing lymph node metastasis if they did not chew betel nut. The interaction between betel quid chewing and rs32000401 polymorphisms on OSCC progression should be further investigated in future work.

**Figure 3:**
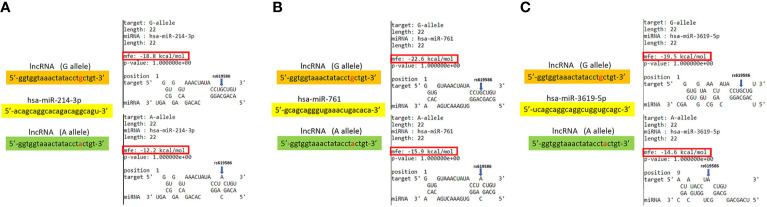
Prediction of potential binding between metastasis-associated lung adenocarcinoma transcript 1 (MALAT1) and its interacting microRNAs. The schematic diagram exhibits hybridization between MALAT1 harboring the rs619586 A or G allele and miR-214 **(A)**, miR-761**(B),** or miR-3619-5p **(C)**. The single-nucleotide polymorphism (SNP) rs619586 A allele reduced the binding affinities with these miRNAs. The positions of rs619586 SNPs are indicated by blue arrows. MFE, minimum free energy.

**Figure 4:**
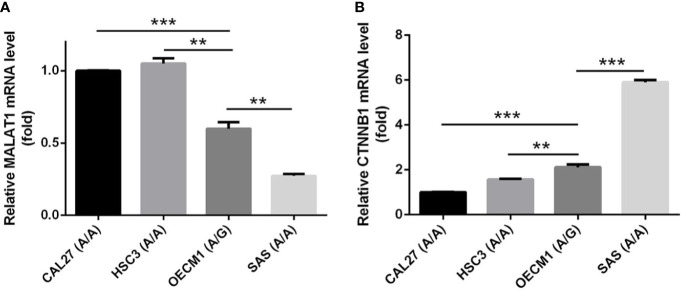
The genotypes of *MALAT1* rs619586 and mRNA levels of *MALAT1* **(A)** and *CTNNB1* **(B)** in four oral squamous cell carcinoma (OSCC) cells (CAL27, HSC3, OECM1, and SAS) were detected by TaqMan SNP Genotyping Assay and RT-qPCR, respectively. Quantitative results of *MALAT1* and *CTNNB1* mRNA levels were adjusted to *GAPDH* mRNA levels. Values are presented as the mean ± standard deviation (SD) of three independent experiments. **p < 0.01 and ***p < 0.001 compared to indicated mRNA levels in OECM1 cells.

**Figure 5:**
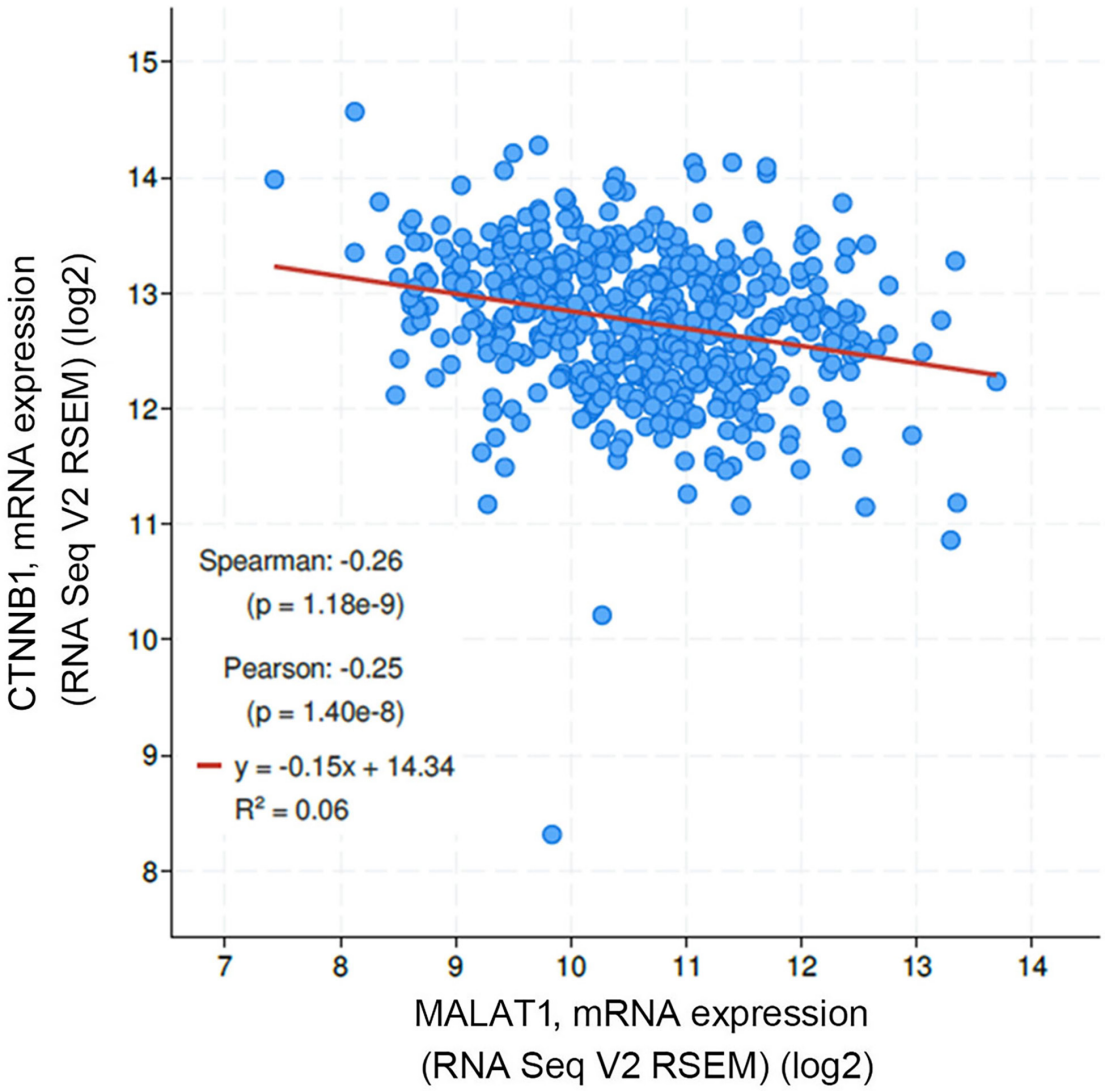
Correlation analysis of TCGA head and neck squamous cell carcinoma (HNSCC) database (TCGA, PanCancer Atlas) using the cBioPortal showed an inverse correlation of MALAT1 with β-catenin (CTNNB1) mRNA levels.

In addition to rs3200401, OSCC patients harboring at least one polymorphic G allele of MALAT1 rs619586 had a higher frequency of developing advance clinical and tumor T stages, and this phenomenon was further strengthened in the betel quid-chewing subgroup. Similar to our results, Wang et al. indicated that patients with differentiated thyroid carcinoma (DTC) carrying the AG/GG genotypes of the MALAT1 rs619586 polymorphism exhibited higher tumor grades and shorter survival times compared to AA-genotype patients (35). Interestingly, most previous studies indicated that the G allele of rs619586 could significantly decrease MALAT1 expression in different cancer types such as DTC (35), breast cancer (20), and papillary thyroid cancer (PTC) (36). MALAT1 was reported to play an oncogenic role in many solid tumors including OSCC (10, 11), and we actually observed that MALAT1 expression was prone to be upregulated in HNSCC from the Western society and significantly upregulated in OSCC specimens from Taiwanese cohort, especially in patients who chewed betel quid. This discrepancy may be due to the differences of genetic backgrounds and pathogenic mechanisms in head and neck cancer between Asian and Western societies. For example, chewing betel quid is the strongest risk factors for causing OSCC in East Asian population (24). In contrast, the tobacco smoking is the most-established risk factor for head and neck cancer in the Western society (37), suggesting the various pathogenic mechanisms in the different ethnic cohorts may lead the different MALAT1 expression status. Herein, our results showed that the G allele of rs619586 might trigger lower MALAT1 expression but was correlated with advance clinical and tumor T stages. Recently, a growing number of reports suggested that MALAT1 acts as a ‘sponge’ to bind specific miRNAs and upregulate miRNAs’ targets to modulate cancer progression. For example, MALAT1 promoted OSCC development *via* sponging miR-125b and subsequently upregulating miR-125b’s target gene, *STAT3* (10). Moreover, MALAT1 induced OSCC invasion by negatively regulating miR-101 and upregulating miR-101’s target gene, *EZH2 (* 11). Large numbers of SNPs were predicted to have potential impacts on miRNA-lncRNA interactions (38). For example, the G variant of the rs619586 SNP was reported to interact with mir-214 and upregulate its target gene, *CTNNB1* (encoding β-catenin) to promote proliferation of DTC (35). Actually, miR-214 and CTNNB1 were respectively reported as having tumor suppressive and oncogenic roles in OSCC (39, 40). By estimating the MFE of the miRNA/MALAT1 duplex, we observed that the A allele of rs619586 led to a far worse energy of miR-214 hybridization of −12.2 kcal/mole than did the G allele (−18.8 kcal/mole) (**Figure 3A**), suggesting that the G allele of MALAT1 rs619586 enhances miR-214 binding to MALAT1. We next examined rs619586 genotypes of four OSCC cell lines (CAL27, HSC3, OECM1, and SAS) and found that OECM1 cells carrying AG genotype of rs619586 respectively expressed lower MALAT1 and higher CTNNB1 levels compared to CAL27 and HSC3 cells carrying AA genotype of rs619586 (**Figures 4A, B**). Furthermore, we analyzed 523 HNSCC human samples that were retrieved from TCGA by using the cBioportal platform and observed that MALAT1 expression inversely correlated with CTNNB1 (**Figure 5**). These phenomena suggested that although the G allele of rs619586 may cause a decrease in MALAT1 levels, this MALAT1 SNP can sponge miR-214 to upregulate CTNNB1 and further promote OSCC progression. In addition to miR-214, the association between miR-761 or miR-3619-5p with the G variant of rs619586 SNP was also predicted by BiBiServ2 RNAhybrid (https://bibiserv.cebitec.uni-bielefeld.de/rnahybrid), and the G allele had better binding stability to these two miRNAs (**Figures 3B, C**). Interactions of different lncRNAs with miR-761 or miR-3619-5p were reported to promote progression or chemoresistance of cancers. For example, the lncRNA, HOXA11-AS, was reported to be a molecular sponge that binds to miR-761 and subsequently upregulates miR-761’s target of *tripartite motif-containing protein 29* (*TRIM29*) to promote progression of PTC (41). Moreover, the lncRNA, KCNQ1OT1, was reported to confer chemoresistance in gliomas *via* sponging miR-761 and subsequently upregulating peripheral interface module 1 (PIM1) (42). The exosomal lncRNA, HEIH, was shown to confer cisplatin resistance in OSCC by sponging miR-3619-5p and upregulating hepatoma-derived growth factor (HDGF) (43). According to those results, we suggest that MALAT1 might sponge miR-214, miR-761, or miR-3619-5p to promote OSCC development, and MALAT1 rs619586 SNPs may influence the interaction of MALAT1 with these miRNAs.

## Conclusions

In conclusion, we first identified the diverse allelic effects of MALAT1 SNPs (rs3200401 and rs619586) which contribute to the susceptibility and clinicopathologic development of OSCC in a Taiwanese population. In addition, the combined effect of MALAT1 SNPs (rs3200401 and rs619586) with betel nut chewing causally contributes to the development of OSCC and this phenomenon was also observed in OSCC patients with the smoking or drinking habit. Furthermore, the rs619586 G variant may enhance the binding of miR-214, miR-761, or miR-3619-5p to MALAT1 to promote OSCC progression. However, these issues should be further confirmed in future studies, and downstream targets of these miRNAs in regulating OSCC progression also need to be further investigated.

## Data Availability Statement

The original contributions presented in the study are included in the article/**Supplementary Material**. Further inquiries can be directed to the corresponding authors.

## Ethics Statement

This study was approved (no. CS15125) by the Institutional Review Board of Chung Shan Medical University Hospital. The patients/participants provided their written informed consent to participate in this study.

## Author Contributions

Y-FD and M-HC contributed to conception, drafted the manuscript, and critically revised the manuscript. S-FY, C-WL, and Y-CW contributed to conception, performed the experiments, and analyzed data. Y-CY, C-YC, Y-FL, L-CC, and W-MC performed the in silico data exploration, integration, and analysis. All authors contributed to the article and approved the submitted version.

## Funding

This work was supported by grants from Wan Fang Hospital, Taipei Medical University (110-wf-swf-06 to Y-CW and M-HC; 110-wf-phd-03 to Y-FD) and the TMU Research Center of Cancer Translational Medicine from The Featured Areas Research Center Program within the framework of the Higher Education Sprout Project by the Ministry of Education in Taiwan (to M-HC).

## Conflict of Interest

The authors declare that the research was conducted in the absence of any commercial or financial relationships that could be construed as a potential conflict of interest.
