# Supplementary material for: Combined Impacts of Genetic Variants of Long Non-Coding RNA MALAT1 and the Environmental Carcinogen on the Susceptibility to and Progression of Oral Squamous Cell Carcinoma
Source: Front Oncol. 2021 Jun 29;11:684941. doi: 10.3389/fonc.2021.684941 (PMC8276129; doi:10.3389/fonc.2021.684941)
Supplement: Supplementary file 1 [file DataSheet_1.pdf]

Supplementary Table 1. Adjusted odds ratios (AORs) and 95% confidence intervals (CIs) of clinical statuses associated with genotypic frequencies of *MALAT1* rs619586 in male oral cancer patients who smoked (n=1140).

Variable			AOR (95% CI)	*p* value
	**Clinical Stage**			
**rs619586**	Stage I+II<br>(n=536) (%)	Stage III+IV<br>(n=604) (%)		
AA	464 (86.6%)	498 (82.5%)	1.00	
AG+GG	72 (13.4%)	106 (17.5%)	**1.387 (1.001-1.921)**	***p*=0.049***
	**Tumor size**			
**rs619586**	≦ T2<br>(n=596) (%)	> T2<br>(n=544) (%)		
AA	518 (86.9%)	444 (81.6%)	1.00	
AG+GG	78 (13.1%)	100 (18.4%)	**1.494 (1.082-2.062)**	***p*=0.015***
	**Lymph node metastasis**			
**rs619586**	No<br>(n=754) (%)	Yes<br>(n=386) (%)		
AA	636 (84.4%)	326 (84.5%)	1.00	
AG+GG	118 (15.6%)	60 (15.5%)	1.003 (0.714-1.409)	*p*=0.986
	**Metastasis**			
**rs619586**	M0<br>(n=1131) (%)	M1<br>(n=9) (%)		
AA	953 (84.3%)	9 (100.0%)	1.00	
AG+GG	178 (15.7%)	0 (0.0%)	-	-
	**Cell differentiated grade**			
**rs619586**	≦Grade I<br>(n=168) (%)	>Grade I<br>(n=972) (%)		
AA	142 (84.5%)	820 (84.4%)	1.00	
AG+GG	26 (15.5%)	152 (15.6%)	1.019 (0.648-1.603)	*p*=0.935

Cell differentiate grade: grade I: well differentiated; grade II: moderately differentiated; grade III: poorly differentiated.

The adjusted odds ratio (AOR) with their 95% confidence intervals were estimated by multiple logistic regression models after controlling for age, betel quid chewing, and alcohol drinking. * *p* value < 0.05 as statistically significant.

Supplementary Table 2. Adjusted odds ratios (AORs) and 95% confidence intervals (CIs) of clinical statuses associated with genotypic frequencies of *MALAT1* rs619586 in male oral cancer patients who drank (n=638).

Variable			AOR (95% CI)	*p* value
	**Clinical Stage**			
**rs619586**	Stage I+II (n=295) (%)	Stage III+IV (n=343) (%)		
AA	257 (87.1%)	276 (80.5%)	1.00	
AG+GG	38 (12.9%)	67 (19.5%)	**1.652 (1.068-2.558)**	***p*=0.024***
	**Tumor size**			
**rs619586**	≦ T2 (n=337) (%)	> T2 (n=301) (%)		
AA	292 (86.7%)	241 (80.1%)	1.00	
AG+GG	45 (13.3%)	60 (19.9%)	**1.576 (1.030-2.411)**	***p*=0.036***
	**Lymph node metastasis**			
**rs619586**	No (n=409) (%)	Yes (n=229) (%)		
AA	345 (84.4%)	188 (82.1%)	1.00	
AG+GG	64 (15.6%)	41 (17.9%)	1.162 (0.752-1.797)	*p*=0.500
	**Metastasis**			
**rs619586**	M0 (n=631) (%)	M1 (n=7) (%)		
AA	526 (83.4%)	7 (100.0%)	1.00	
AG+GG	105 (16.6%)	0 (0.0%)	-	-
	**Cell differentiated grade**			
**rs619586**	≦Grade I (n=90) (%)	>Grade I (n=548) (%)		
AA	76 (84.4%)	457 (83.4%)	1.00	
AG+GG	14 (15.6%)	91 (16.6%)	1.022 (0.551-1.897)	*p*=0.944

Cell differentiate grade: grade I: well differentiated; grade II: moderately differentiated; grade III: poorly differentiated.

The adjusted odds ratio (AOR) with their 95% confidence intervals were estimated by multiple logistic regression models after controlling for age, betel quid chewing, and cigarette smoking. * *p* value < 0.05 as statistically significant.

Supplementary Table 3. Adjusted odds ratios (AORs) and 95% confidence intervals (CIs) of clinical statuses associated with genotypic frequencies of *MALAT1* rs3200401 in male oral cancer patients who smoked (n=1140).

Variable			AOR (95% CI)	*p* value
	**Clinical Stage**			
**rs3200401**	Stage I+II (n=536) (%)	Stage III+IV (n=604) (%)		
CC	371 (69.2%)	436 (72.2%)	1.00	
CT+TT	165 (30.8%)	168 (27.8%)	0.864 (0.669-1.117)	*p*=0.264
	**Tumor size**			
**rs3200401**	≦ T2 (n=596) (%)	> T2 (n=544) (%)		
CC	420 (70.5%)	387 (71.1%)	1.00	
CT+TT	176 (29.5%)	157 (28.9%)	0.969 (0.750-1.252)	*p*=0.809
	**Lymph node metastasis**			
**rs3200401**	No (n=754) (%)	Yes (n=386) (%)		
CC	529 (70.2%)	278 (72.0%)	1.00	
CT+TT	225 (29.8%)	108 (28.0%)	0.914 (0.696-1.201)	*p*=0.519
	**Metastasis**			
**rs3200401**	M0 (n=1131) (%)	M1 (n=9) (%)		
CC	802 (70.9%)	5 (55.6%)	1.00	
CT+TT	329 (29.1%)	4 (44.4%)	2.003 (0.532-7.545)	*p*=0.305
	**Cell differentiated grade**			
**rs3200401**	≦Grade I (n=168) (%)	>Grade I (n=972) (%)		
CC	132 (78.6%)	675 (69.4%)	1.00	
CT+TT	36 (21.4%)	297 (30.6%)	**1.625 (1.096-2.409)**	***p*=0.016***

Cell differentiate grade: grade I: well differentiated; grade II: moderately differentiated; grade III: poorly differentiated.

The adjusted odds ratio (AOR) with their 95% confidence intervals were estimated by multiple logistic regression models after controlling for age, betel quid chewing, and alcohol drinking. * *p* value < 0.05 as statistically significant.

Supplementary Table 4. Adjusted odds ratios (AORs) and 95% confidence intervals (CIs) of clinical statuses associated with genotypic frequencies of *MALAT1* rs3200401 in male oral cancer patients who drank (n=638).

Variable			AOR (95% CI)	*p* value
	**Clinical Stage**			
**rs3200401**	Stage I+II (n=295) (%)	Stage III+IV (n=343) (%)		
CC	199 (67.5%)	248 (72.3%)	1.00	
CT+TT	96 (32.5%)	95 (27.7%)	0.798 (0.568-1.123)	*p*=0.196
	**Tumor size**			
**rs3200401**	≦ T2 (n=337) (%)	> T2 (n=301) (%)		
CC	229 (68.0%)	218 (72.4%)	1.00	
CT+TT	108 (32.0%)	83 (27.6%)	0.806 (0.573-1.135)	*p*=0.217
	**Lymph node metastasis**			
**rs3200401**	No (n=409) (%)	Yes (n=229) (%)		
CC	284 (69.4%)	163 (71.2%)	1.00	
CT+TT	125 (30.6%)	66 (28.8%)	0.925 (0.647-1.322)	*p*=0.668
	**Metastasis**			
**rs3200401**	M0 (n=631) (%)	M1 (n=7) (%)		
CC	442 (70.1%)	5 (71.4%)	1.00	
CT+TT	189 (29.9%)	2 (28.6%)	0.960 (0.184-5.013)	*p*=0.961
	**Cell differentiated grade**			
**rs3200401**	≦Grade I (n=90) (%)	>Grade I (n=548) (%)		
CC	70 (78.6%)	377 (68.8%)	1.00	
CT+TT	20 (21.4%)	171 (31.2%)	**1.609 (0.946-2.736)**	***p*=0.008**

Cell differentiate grade: grade I: well differentiated; grade II: moderately differentiated; grade III: poorly differentiated.
The adjusted odds ratio (AOR) with their 95% confidence intervals were estimated by multiple logistic regression models after controlling for age, betel quid chewing, and cigarette smoking. * *p* value < 0.05 as statistically significant.
